# Supplementary material for: In Vitro Erythropoiesis at Different pO2 Induces Adaptations That Are Independent of Prior Systemic Exposure to Hypoxia
Source: Cells. 2022 Mar 23;11(7):1082. doi: 10.3390/cells11071082 (PMC8997720; doi:10.3390/cells11071082)
Supplement: Supplementary file 1 [file cells-11-01082-s001.zip › cells-1629370-supplementary.pdf]

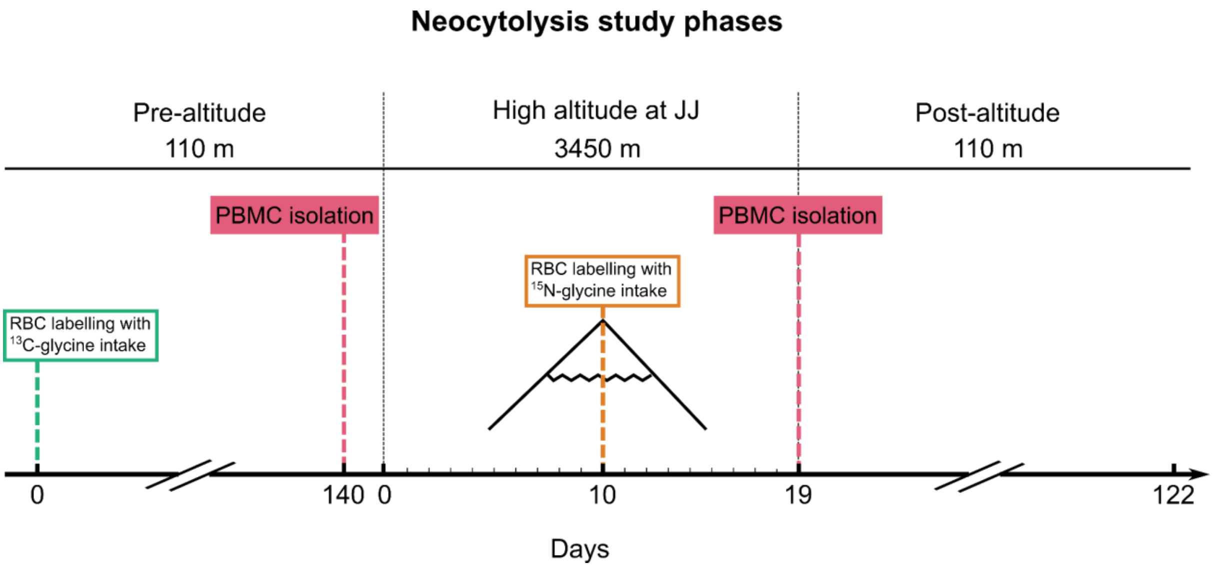

Figure S1. Neocytolysis study timeline, divided into three phases: in the pre-altitude phase (day 0) RBC were age-cohort labeled with the isotope ^{13}C-glycine; a second age-cohort labeling with ^{15}N was performed at high altitude (day 10). RBC decay was regularly measured by isotope ratio mass spectrometry haem ($^{13}C/^{12}C$ in the pre-altitude phase and $^{15}N/^{14}N$ during high altitude and post-altitude phases) to reveal any difference in RBC lifespan between the two cohorts that could demonstrate the existence of neocytolysis. PBMC collection for *in vitro* erythropoiesis was performed at day 140 pre-altitude and day 19 high altitude (JJ samples). For more information on the study, see Klein et al. [11].

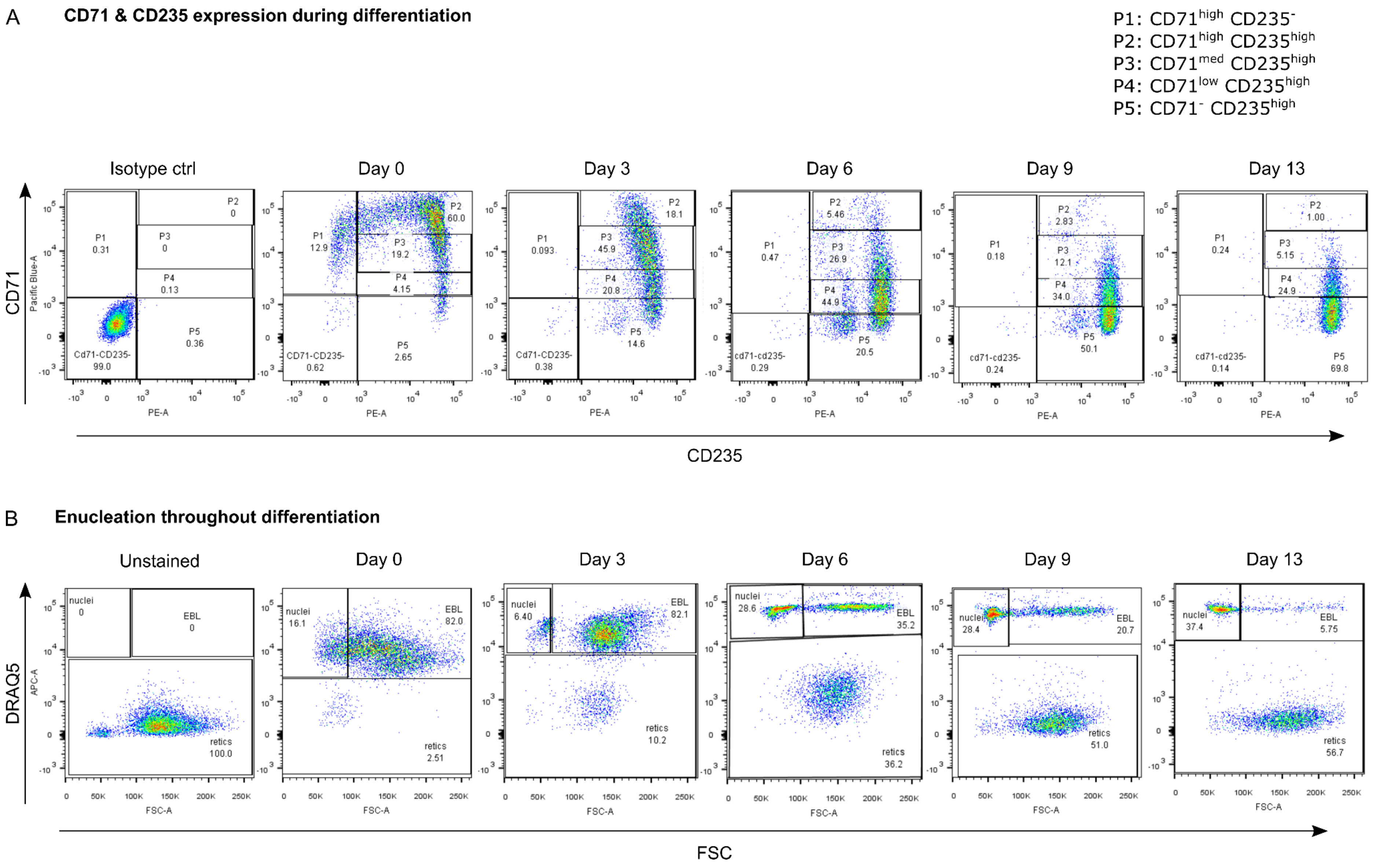
A
CD71 & CD235 expression during differentiation
P1: CD71^high CD235^-
P2: CD71^high CD235^high
P3: CD71^med CD235^high
P4: CD71^low CD235^high
P5: CD71^- CD235^high
Isotype ctrl
Day 0
Day 3
Day 6
Day 9
Day 13
CD71
Pacific Blue-A
PE-A
CD235
P1 0.31
P2 0
P3 0
P4 0.13
P5 0.36
Cd71-CD235- 99.0
P1 12.9
P2 60.0
P3 19.2
P4 4.15
P5 2.65
CD71-CD235- 0.62
P1 0.093
P2 18.1
P3 45.9
P4 20.8
P5 14.6
Cd71-CD235- 0.38
P1 0.47
P2 5.46
P3 26.9
P4 44.9
P5 20.5
cd71-cd235- 0.29
P1 0.18
P2 2.83
P3 12.1
P4 34.0
P5 50.1
cd71-cd235- 0.24
P1 0.24
P2 1.00
P3 5.15
P4 24.9
P5 69.8
cd71-cd235- 0.14
B
Enucleation throughout differentiation
Unstained
Day 0
Day 3
Day 6
Day 9
Day 13
DRAQ5
APC-A
FSC-A
FSC
nuclei 0
EBL 0
retics 100.0
nuclei 16.1
EBL 82.0
retics 2.51
nuclei 6.40
EBL 82.1
retics 10.2
nuclei 28.6
EBL 35.2
retics 36.2
nuclei 28.4
EBL 20.7
retics 51.0
nuclei 37.4
EBL 5.75
retics 56.7

Figure S2. Representative flow cytometry dot plot on one donor over differentiation. (A) Population gating of erythroid precursors at different stages of maturation according to the expression of CD71 and CD235. $CD71^-CD235^-$ indicate non-erythroid cells. (B) DRAQ5 nuclear staining vs FSC. Positive events refer to nucleated cells (EBL=erythroblasts) and pyrenocytes (nuclei) and negative events to enucleated reticulocytes.

Donor 1 at 20% O_2

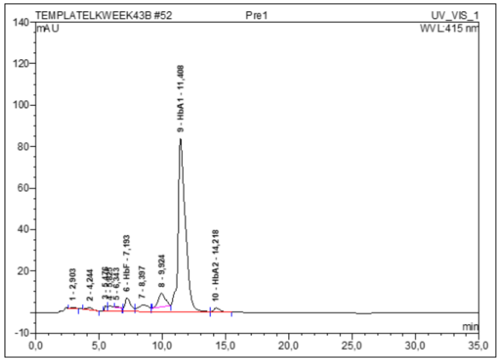

No.	Ret.Time	Peak Name	Rel.Area
	min		%
1	2.90	n.a.	0.46
2	4.24	n.a.	0.76
3	5.48	n.a.	0.78
4	5.83	n.a.	3.35
5	6.34	n.a.	0.20
6	7.19	HbF	4.67
7	8.40	n.a.	4.06
8	9.92	n.a.	5.51
9	11.41	HbA1	78.81
10	14.22	HbA2	1.39
**Total:**			100.00

Donor 1 at 3% O_2

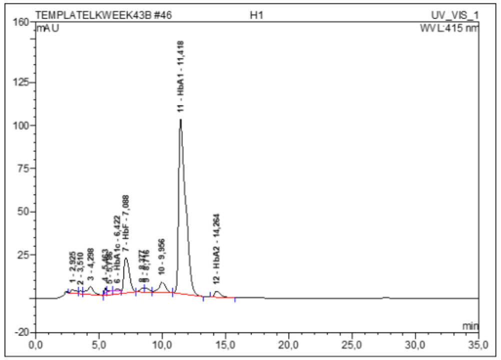

No.	Ret.Time	Peak Name	Rel.Area
	min		%
1	2.92	n.a.	1.18
2	3.51	n.a.	0.59
3	4.30	n.a.	3.16
4	5.46	n.a.	0.49
5	5.79	n.a.	0.12
6	6.42	HbA1c	0.50
7	7.09	HbF	14.03
8	8.38	n.a.	0.92
9	8.72	n.a.	1.12
10	9.96	n.a.	4.10
11	11.42	HbA1	71.70
12	14.26	HbA2	2.08
**Total:**			100.00

Donor 1 at 20% O_2 JJ

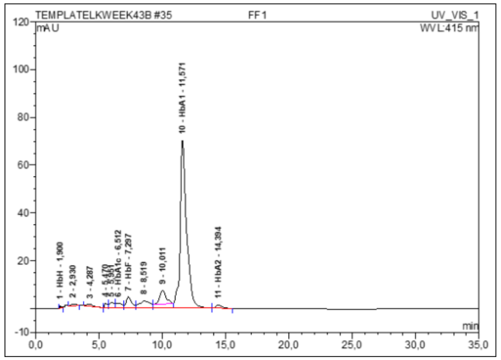

No.	Ret.Time	Peak Name	Rel.Area
	min		%
1	1.90	HbH	0.20
2	2.93	n.a.	0.59
3	4.29	n.a.	0.80
4	5.47	n.a.	0.84
5	5.95	n.a.	1.93
6	6.51	HbA1c	1.86
7	7.30	HbF	4.02
8	8.52	n.a.	5.00
9	10.01	n.a.	6.02
10	11.57	HbA1	77.46
11	14.39	HbA2	1.26
**Total:**			100.00

Figure S3. Representative examples of primary HPLC data. Donor 1 in the three culture conditions.

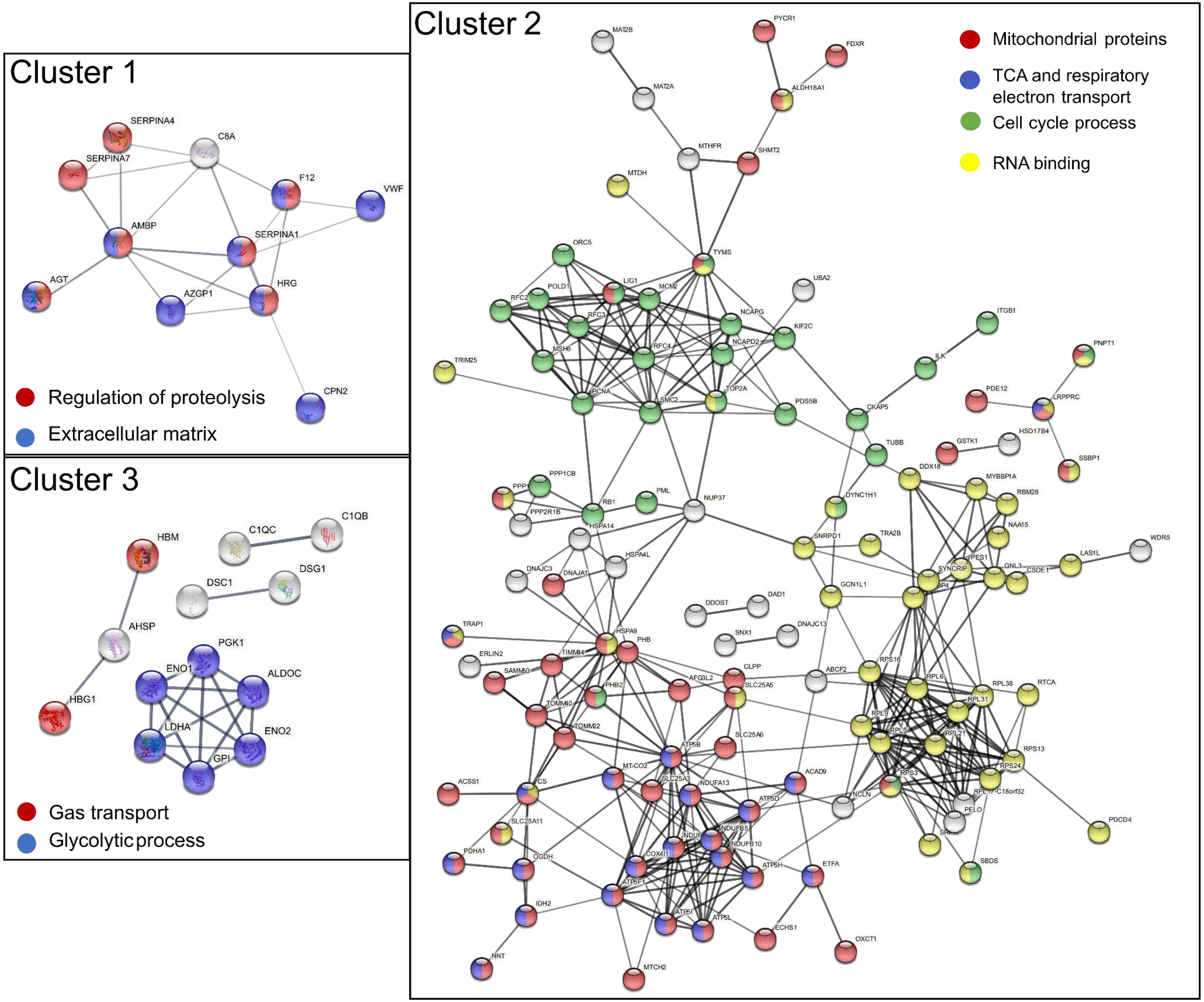

Cluster 1
SERPINA4
C8A
SERPINA7
F12
VWF
AMBP
SERPINA1
AGT
AZGP1
HRG
CPN2
Regulation of proteolysis
Extracellular matrix
Cluster 3
C1QB
C1QC
HBM
DSG1
DSC1
AHSP
PGK1
ENO1
ALDOC
HBG1
LDHA
ENO2
GPI
Gas transport
Glycolytic process
Cluster 2
Mitochondrial proteins
TCA and respiratory electron transport
Cell cycle process
RNA binding
MAT2B
PYCR1
FDXR
MAT2A
ALDH18A1
MTHFR
SHMT2
MTDH
ORC5
TYMS
UBA2
LIG1
POLD1
MCM2
RFC2
RFC3
NCAPG
KIF2C
NCAPD2
RFC4
MSH6
ITGB1
ILK
PNPT1
TRIM25
PCNA
SMC2
TOP2A
PDS5B
CKAP5
PDE12
LRPPRC
HSD17B4
GSTK1
TUBB
SSBP1
DDX18
MYBBP1A
RBM28
PPP1CB
PML
RB1
PPP2R1B
NUP37
DYNC1H1
HSPA14
NAA15
SNRPD1
TRA2B
HSPA4L
PES1
SYNCRIP
GNL3
CSDE1
LAS1L
WDR5
DNAJC3
DNAJA1
GCN1L1
DDOST
DAD1
SNX1
DNAJC13
TRAP1
HSPA9
PHB
ERLIN2
TIMM44
SAMM50
CLPP
AFG3L2
SLC25A5
ABCF2
RPS16
RPL6
RPL38
RTCA
PHB2
TOMM40
RPL31
RPL9
TOMM22
RPL5
RPL21
SLC25A6
ATP5B
RPS13
MT-CO2
RPS24
SLC25A3
NDUFA13
RPS3
ACAD9
ACSS1
CS
NCLN
RPL17-C18orf32
ATP5D
PELO
SLC25A11
NDUFB5
NDUFB10
PDCD4
PDHA1
OGDH
COX4I1
ATP5H
ETFA
SBDS
ATP5F1
IDH2
ATP5I
ATP5L
ECHS1
OXCT1
NNT
MTCH2

Figure S4. STRING analysis [59] of significantly differentially abundant proteins between the 3% and 20% O_2 cultures. Clusters are defined in figure 6D. Proteins are highlighted in different colors according to their functional group.
